# Virtual Hemodynamic Assessment of Coronary Lesions: The Advent of Functional Angiography and Coronary Imaging

**DOI:** 10.3390/jcm13082243

**Published:** 2024-04-12

**Authors:** Sotirios Nikopoulos, Michail I. Papafaklis, Panagiota Tsompou, Antonis Sakellarios, Panagiotis Siogkas, Spyros Sioros, Dimitrios I. Fotiadis, Christos S. Katsouras, Katerina K. Naka, Dimitrios Nikas, Lampros Michalis

**Affiliations:** 1 Department of Cardiology, Medical School, University of Ioannina, 45110 Ioannina, Greece; spsior@yahoo.com (S.S.); cskats@yahoo.com (C.S.K.); anaka@uoi.gr (K.K.N.); dimitrios.nikas@gmail.com (D.N.); lamprosmihalis@gmail.com (L.M.); 2 Cardiology Division, Medical School, University of Patras, 26504 Patras, Greece; m.papafaklis@yahoo.com; 3 Department of Biomedical Research, Institute of Molecular Biology and Biotechnology-FORTH, University Campus of Ioannina, 45115 Ioannina, Greece; panagiotatsompou@gmail.com (P.T.); psiogkas@uoi.gr (P.S.); fotiadis@uoi.gr (D.I.F.); 4 Unit of Medical Technology and Intelligent Information Systems, Department of Materials Science and Engineering, University of Ioannina, 45110 Ioannina, Greece; 5 Department of Mechanical Engineering and Aeronautics, University of Patras, 26504 Rio, Greece; ansakel13@gmail.com

**Keywords:** cardiovascular disease, three-dimensional reconstruction, computational fluid dynamics, virtual functional assessment, medical imaging, angiography

## Abstract

The fractional flow reserve (FFR) is well recognized as a gold standard measure for the estimation of functional coronary stenosis. Technological progressions in image processing have empowered the reconstruction of three-dimensional models of the coronary arteries via both non-invasive and invasive imaging modalities. The application of computational fluid dynamics (CFD) techniques to coronary 3D anatomical models allows the virtual evaluation of the hemodynamic significance of a coronary lesion with high diagnostic accuracy. Methods: Search of the bibliographic database for articles published from 2011 to 2023 using the following search terms: invasive FFR and non-invasive FFR. Pooled analysis of the sensitivity and specificity, with the corresponding confidence intervals from 32% to 94%. In addition, the summary processing times were determined. Results: In total, 24 studies published between 2011 and 2023 were included, with a total of 13,591 patients and 3345 vessels. The diagnostic accuracy of the invasive and non-invasive techniques at the per-patient level was 89% (95% CI, 85–92%) and 76% (95% CI, 61–80%), respectively, while on the per-vessel basis, it was 92% (95% CI, 82–88%) and 81% (95% CI, 75–87%), respectively. Conclusion: These opportunities providing hemodynamic information based on anatomy have given rise to a new era of functional angiography and coronary imaging. However, further validations are needed to overcome several scientific and computational challenges before these methods are applied in everyday clinical practice.

## 1. Introduction

Coronary artery disease (CAD) is the main cause of morbidity and mortality around the world [1]. Coronary revascularization by percutaneous coronary intervention (PCI) restores the epicardial flow and aims at relieving angina and myocardial ischemia. Recent studies have revealed that in patients with chronic coronary artery disease, the option of whether to perform PCI in moderate lesions is difficult, as both visual assessment and quantitative coronary analysis (QCA) have revealed low correlation with the functional stenosis severity [2,3]. Therefore, the need to evaluate both the functional and anatomical severity of obstructive CAD, especially when intermediate stenosis is present, has prompted the adoption of invasive functional indices, principally the fractional flow reserve. The FFR has enabled interventional specialists to precisely decide which lesions are functionally significant and thus may lead to ischemia and future adverse events [4,5]. The clinical importance of the FFR assessment measured by an intracoronary pressure wire has been acknowledged in the international guidelines and long-term data have shown improved outcomes with FFR-guided decision-making. During the last decade, the use of the FFR has slowly increased from 14.8% to 18.5% in patients with angiographically intermediate stenosis [6]. However, its use differs widely across the catheterization laboratories globally. This distinction may be related to the invasive nature of FFR evaluation, the prolongation of the cardiac catheterization, the use of hyperemic agents and the high equipment and pharmacologic costs [7]. To overcome these limitations, new methodologies for assessing physiological indices based on fluid mechanics principles have been advanced to reduce the interventional feature of the FFR methodology estimation. The progress of technology has also allowed the wide-ranging application of computational fluid dynamics techniques to coronary imaging, thereby enabling the accurate estimation of the severe occlusions of coronary arterial lesions [8]. During the last decade, these techniques have been applied to both non-invasive (i.e., coronary computed tomography angiography [CCTA]) and invasive (angiography or intravascular imaging in the catheterization laboratory) coronary imaging datasets. All these methods make various assumptions for computing the equivalent FFR values and demonstrate high diagnostic performance and discriminatory power. The current review summarizes the computational models based on invasive and non-invasive imaging, the application of virtual indices in clinical studies and their performance against the gold standard FFR, and it discusses the future steps and opportunities for incorporating virtual functional assessment into the management strategy of high cardiovascular risk patients.

## 2. Fluid Mechanics and Computational Fluid Dynamics

**Figure 1:**
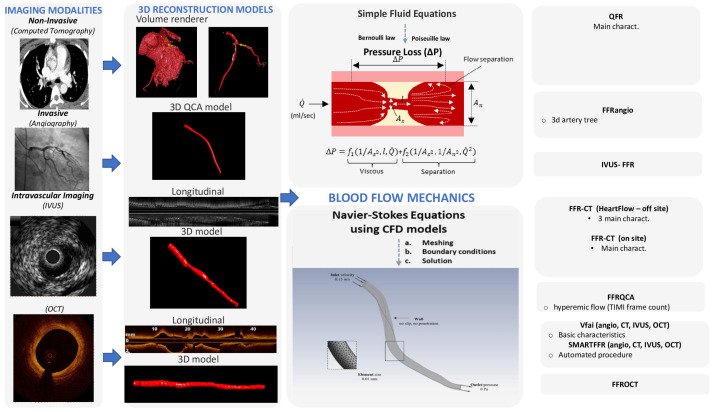
Technological progressions in image processing have empowered the reconstruction of three-dimensional models of the coronary arteries via both non-invasive imaging modalities, such as coronary computed tomography angiography (CCTA), and invasive coronary angiography or intravascular imaging. The application of computational fluid dynamics (CFD) techniques to coronary 3D anatomical models allows the virtual evaluation of the hemodynamic significance of a coronary lesion.

To assess the hemodynamic conditions in the cardiovascular system, it is required to take into account the fluid properties and the blood flow characteristics and then to apply the universal laws of fluid mechanics [8,9]. The blood flow in the circulation system is considered as incompressible and is characterized by pulsatility. The arterial walls are distensible, and coronary arteries are displaced and distorted because of the heart beating. The main fluid properties are the density and viscosity, which can either be considered as constant (i.e., Newtonian fluid) for simplicity or may be more accurately described by an apparent viscosity value that changes non-linearly due to the applied stress (i.e., non-Newtonian fluid) [9]. The impact of the pressure cut within the stenosis occurs mostly because of the frictional losses arising from the entrance variations (entrance effects) and the kinetic energy loss derived from the lumen expansion that yields zones of recirculation. The calculation of the pressure loss can be estimated according to the laws of Bernoulli and Poiseuille and is highly associated with various factors, including the culprit lesions, the area and the range of the stenosis, the flow, and the coefficients of the expansion loss [9]. In addition, each stenosis characteristic, including the morphology and eccentricity of the stenotic lumen, along with the arterial curvature of the stenotic segment, has a tremendous impact on the translational hemodynamics [9]. The computational complexity of the mathematical solutions for the blood flow in the human coronary arteries is large and is affected by the three-dimensional (3D) deformable topology and the pulsatile blood flow and limited by certain assumptions (e.g., the Bernoulli equation is valid for inviscid flow, the Poiseuille equation is valid for steady flow). The solution for the accurate calculation of the coronary blood flow is given by the equations of fluid dynamics, Navier–Stokes equations, which are solved for the unknown pressure that varies according to the position and time and for the three components of blood velocity. Special conditions must be applied to solve the blood flow equations (e.g., steady or pulsatile flow in an idealized cylindrical geometry). Realistic models of the human coronary arteries require the simultaneous solution of a huge amount of nonlinear partial differential equations and a numerical method to obtain a solution for the velocity and pressure at a finite number of points [10]. For a cardiac cycle, the above procedure is repeated for thousands of time intervals. The computational fluid dynamics (CFD) methods are defined as the numerical methods for fluid dynamics problems. Three key components that together make up the necessary framework are required for developing the CFD models: (i) 3D arterial models derived from medical images, (ii) numerical methods for solving the 3D Navier–Stokes equations, and (iii) the application of the initial and boundary conditions [11]. Precise 3D reconstructed coronary artery lesions have been derived from the existing imaging modalities. This rapid progress has enabled the development of the CFD models that allow blood flow simulation in realistic geometries. To perform numerical simulations, three main steps have to be accomplished: (i) the pre-processing stage, (ii) the solver stage, and (iii) the post-processing stage [12]. The pre-processing stage involves the description of the fluid construction with a mesh solver, where the 3D geometry is divided into numerous finite elements. Then, the flowing substance properties and physics of the circulated blood are determined in order to puzzle out the defined mathematical model. Blood is assumed to have a density of approximately 1050 kg/m^3^ and a viscosity of 3.5 × 10^−3^ Pa·s following either the Newtonian law (i.e., constant value) or a non-Newtonian model of viscosity. At the inlet of the artery, stable flow can be applied or, alternatively, a flow waveform to mimic the physiological pulsatile flow in coronary arteries. A no-slip condition and no penetration are applied at the walls and a zero pressure profile is commonly applied at the outlet. The second stage involves the modeling of mathematical calculations to solve the equations of fluid dynamics at the nodes of the finite elements. Finally, after the problem is solved, the last stage involves the extraction and analysis of all the hemodynamic parameters of interest [12]. The pressure gradient can be computed in 3D reconstructed patient-specific arterial models by the ratio of the computed pressure distal to the stenosis and the pressure at the inlet of the 3D model, which corresponds to the aortic pressure [13]. Consequently, the calculation of the pressure gradient under simulated resting or hyperemic (i.e., FFR) conditions depends on the boundary conditions (e.g., resting or hyperemic flow and distal pressure/microcirculatory resistance) applied in the CFD model (Figure 1). 

## 3. Virtual FFR Based on Non-Invasive Imaging 

Progressions in image modalities have allowed the 3D coronary reconstruction of the coronary tree and the recognition of the culprit lesions via non-invasive image modalities like computed coronary tomography angiography (CCTA). These developments have allowed the integration of 3D CT-derived anatomical reconstructions with techniques for estimating the FFR based on CFD (computational fluid dynamics). Lately, a series of studies have proposed that the assessment of the CCTA-based FFR (FFR-CT) might increase the diagnostic accuracy of CCTA. Most of these studies validated its high diagnostic accuracy and revealed its superiority against stenosis assessment by only CCTA, particularly in terms of the improved specificity and positive predictive value.

### 3.1. FFRCT^®^ by HeartFlow: Offsite Computations

**Figure 2:**
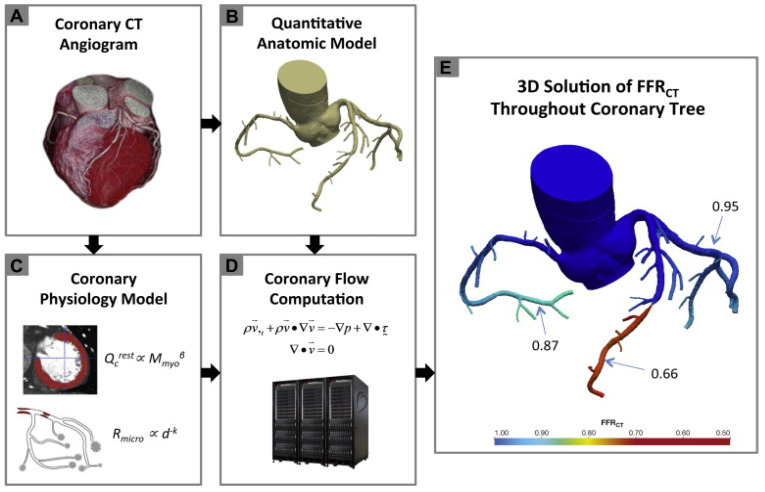
Schematic presentation of the FFRCT analysis [16]. Principles of computational fluid dynamics combine with advances in computing power, numerical methods, and imaged-based modeling. This figure is adapted from Gaur et al. [16] with permission of Elsevier. Full color available online.

The three initial prospective studies, DISCOVER-FLOW (2011) [14], DeFACTO (2012) [15], and HeartFlow^®^ NXT (2015) [16,17], used the invasive FFR as the referral guidance and applied the same cut-off for lesion significance (≤0.8), providing proof that this method can be applied in daily practice. CT angiograms were sent to a central core laboratory (Harbor UCLA Medical Center, Los Angeles, California, USA) for blinded interpretation using an 18-segment coronary model. Three-dimensional models of the coronary anatomy were reconstructed with custom methods applied to CCTA data for simulation of the arterial flow and pressure. The FFRCT^®^ methodology is based on three key theories. The first is that the arterial supply crosses the myocardial demand at rest, the second is that the resistance of the microvascular circulation at rest is inversely but not linearly proportional to the dimension of the feeding vessel and the last is that the microcirculation reacts predictably to the maximum hyperemic condition in case studies with no coronary stenosis. Initially, the FFRCT^®^ study, the DISCOVER FLOW study [14], included 103 patients (159 vessels) with an occlusion in a major coronary lesion and the diagnostic performances of FFRCT^®^ and CCTA were compared. For the single vessel analysis, the accuracy, specificity, sensitivity, positive predictive value, and negative predictive value for FFRCT^®^ and CCTA were 84, 88, 82, 74, and 92%, respectively. This study crossed its primary end point to reveal a relative upgrade in diagnostic accuracy of ≥25% for FFRCT^®^ when compared with CCTA stenosis. A year later, a larger multicenter study [15] (DeFACTO) concluded 615 vessels, where CT, FFRCT^®^, invasive coronary angiography, and the invasive FFR was applied. Notably, 150 vessels of moderate stenosis by CT, defined as 30% to 69% stenosis, were detected. For vessels of moderate stenosis, the severity specificity, sensitivity, accuracy, positive predictive value and negative predictive value of FFRCT were 67%, 74%, 71%, 41%, and 90%, whereas the specificity, sensitivity, accuracy, positive predictive value and negative predictive value of CT stenosis were 72%, 34%, 63%, 27%, and 78%. FFRCT^®^, in comparison with CT stenosis, revealed a superior distinction and provided high diagnostic accuracy for the detection of ischemia where segments with intermediate stenosis were involved. Particularly, the above results suggest the potential of FFRCT to functionally rule out intermediate ischemic lesions. The diversity among the current study and the previous report [14] was that the present data comprise all the stenoses between 30% and 69% stenosis severity. Furthermore, the current data were derived from a large perspective, multicenter study. The other multicenter study [15] (NXT) showed that FFRCT highly upgraded both the per-vessel specificity and positive predictive value (60 to 86% and 33 to 67%, respectively), and the specificity and positive predictive value (32% to 84% and 40% to 65%, respectively) compared with the CCTA characteristic curve, which increased from 0.81 to 0.90 (*p* = 0.0008). Similarly, in 235 patients with a degree of stenosis from 30% to 70%, the specificity and positive predictive value increased from 32% to 79% and 37% to 63%, respectively, per patient. In the NXT study, improvements to the physiological models of microcirculatory resistance were applied, which were confirmed in a retrospective evaluation using data from the DISCOVER-FLOW and DeFACTO studies. In this study, an advanced proprietary software was used to update the proprietary optimizing automation, image quality assessment and image segmentation (Figure 2). 

### 3.2. FFRCT by Other Groups: Onsite Studies

**Figure 3:**
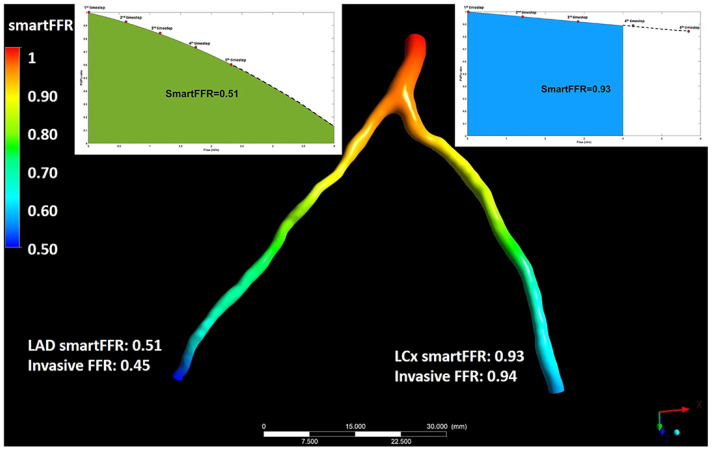
Illustration depicting the simultaneous SmartFFR calculation process for the two main branches of the left coronary vasculature (i.e., LAD and LCx). The dashed line after the last simulation timestep at the LAD branch was used to extrapolate the curve to reach the 4 mL/s mark, whereas for the LCx branch, the curve was interpolated to limit the curve to the 4 mL/s mark, respectively. This figure is adapted from Siogkas et al. [27] with permission of Frontiers. Full color available online.

The aforementioned studies required transferring patient image data to an external core laboratory [18]. The calculation and transfer process remains time consuming (around 1 to 4 h) and is less suitable for prompt clinical decision-making, which obviously limits the practical utility. As a result, less demanding computational approaches using regular workstations have been developed [19]. This allows near real-time FFR estimation using workstations at the point of care. Along with the FFRCT^®^ reports, there are several studies using CFD algorithms based on local workstations. Renker et al. [20], performed a retrospective study with less than 60 min of analysis time and an FFR of <0.80 as the gold standard. In a per lesion analysis, the sensitivity was 85% and 94%, the specificity was 85% and 84%, and the positive and negative predictive values of FFR-CT were 71% and 93%, respectively. On a per-patient basis, the characteristic curve was of borderline superiority to CTA alone (0.91 versus 0.78, *p* = 0.078). A single-center workstation-based study with a 20 min processing time analyzing 96 lesions in 90 patients with an invasive FFR of <0.80 as the gold standard was performed by Kruk et al. [21]. They reported 76% sensitivity, 72% specificity, 67% positive predictive value, and 80% negative predictive value compared with 100% sensitivity, 2% specificity, 43% positive predictive value, and 100% negative predictive value for CTA alone. The per-vessel accuracy of FFR-CT was beneath than that of DISCOVER-FLOW (84%) or NXT (86%), but higher than in DeFACTO (69%). The main pitfalls of these studies [20,21] were the limited number of patients and their single-center character, which is substantially methodologically inferior to the previous multicenter studies [14,15,16]. FFRCT^®^ (HeartFlow^®^) is based on 3D geometric modeling and computationally intense blood flow analysis, which require offsite supercomputing power, and the boundary conditions are determined by allometric scaling laws and assumptions regarding microvascular resistance. However, Ko et al. [22] presented an alternative technique for FFR-CT with the borderline physics exported from anatomic deformation of coronary lumen and aorta and reduced order or one-dimensional fluid modeling. An increased positive predictive value (74% vs. 60%) and specificity (87% vs. 74%) for FFR-CT than for CCTA alone were observed. This novel approach was reported to require a short processing time (30 min) using a standard desktop computer. Smaller pilot studies investigated the onsite feasibility of ischemic coronary lesion detection. Donnelly et al. [23] evaluated the diagnostic accuracy of a new onsite FFRCT toward to the invasively derived FFR as the gold standard and determined whether its diagnostic performance is affected by interobserver variations in lumen segmentation. In this prospective study, 44 patients were enrolled and both CCTA and invasive coronary angiography (ICA) was performed. Expert readers manually adjusted the semi-automated coronary lesion segmentations for effective diameter stenosis (EDS). They concluded that onsite FFR-CT simulation is feasible and the diagnostic performance of the onsite FFR-CT simulation algorithm does not depend on the readers’ semi-automated lumen segmentation adjustments. The additional procedure time was short and acceptable for integration into a clinical service workflow. Another prototype for onsite determination of FFR-CT on a standard personal computer (PC) compared to the invasively measured FFR in patients with suspected CAD was presented by Röther et al. [24]. In a total 71 patients (91 vessels), a cut-off point of ≤0.80 was indicated as a hemodynamic stenosis marker. The average calculation time of FFR-CT was 12.4 ± 3.4 min. After importing the imaging from the installed software, the coronary imaging centerlines were automatically spotted and the coronary lumen was segmented and then the virtual FFR values for the whole epicardial coronary artery system were analyzed. The computation was based on a machine-learning algorithm that was trained to reproduce the results of an established CFD approach with a very low runtime on standard hardware [17]. The most significant upgrade compared to the previously mentioned studies [14,15,16] using onsite workflows for calculating FFR-CT was the faster average entire processing time for the computation of FFR-CT. This is a novel approach where direct comparison between the diagnostic value of FFR-CT analysis versus ICA is presented. Another recent onsite analysis for the direct diagnostic comparison of FFR-CT and ICA with the invasive FFR as the state of the art in patients with intermediate stenosis on CCTA was performed by Wardziak et al. in 2019 [25]. A total of 96 moderate stenoses (50–90%) from 90 cases, with a moderate pre-test probability of coronary artery disease, who underwent coronary CCTA were analyzed. The aforementioned pilot studies showed the feasibility of onsite FFR-CT, which was also shown to have higher discriminatory power compared to QCA, visual ICA, CCTA and visual CCTA. A novel assessment based on the CCTA virtual functional assessment index (vFAI) using an automated in-house developed software was tested on intermediate coronary stenoses (>30% and ≤90%) compared to the invasively measured FFR [26]. In 63 patients (74 vessels) with chest discomfort and an intermediate (20–90%) pre-test likelihood of CAD undergoing CCTA and invasive angiography with FFR calculation, vFAI measurements were applied after 3D reconstruction of the coronary tree and blood simulations utilizing the finite element strategy. The average diversity of the calculations (CT-based vFAI vs. FFR) was 0.03 (SD = 0.033), indicating a short systematic overestimation of the FFR by vFAI. Despite the small overestimation of the FFR compared to the aforementioned studies, the diagnostic precision of the above method was not inferior to them. The analysis time needed was 25 min on average, much lower than that of the present widely used FFR-CT software (FFRCT core laboratory, HeartFlow, Inc., version 1.4, Redwood City, California) [17], and was equivalent to that of the study by Kruk et al. (average of 20 min per case) [21] or of the study by Ko et al. [22] (average of 27 min per case). Another stand-alone computational methodology for non-invasive calculation of the FFR was presented by Siogkas et al. [27]. SmartFFR is based on a transient blood flow simulation and its novelty lies in the fact that it can be effectively applied to arterial bifurcations. Furthermore, it has been released as a stand-alone version [28] and a cloud-based platform, as well. Another advantage is that it offers lies in its rapid execution since it does not require more than a couple of minutes [27]. In a novel meta-analysis aiming to demonstrate the efficiency of this recently presented SmartFFR index [27], a dataset of 167 patients (202 vessels) was used. The SmartFFR was calculated while both 3D vessel reconstruction and blood flow simulations were performed, with an average execution time of seven minutes. In the net dataset, SmartFFR combined the calculated indexes of the invasively derived FFR, yielding sensitivity and specificity of 94.6% and 85.6%, respectively, using a cut-off value 0.83 to identify stenoses with an FFR ≤ 0.80. CCTA offers several advantages with respect to single-photon emission computerized tomography (SPECT) and positron emission tomography (PET), which provide essential non-invasive imaging information about the functional assessment of CAD. A prospective head-to-head comparison of FFR-CT with CCTA, PET, SPECT and perfusion imaging for ischemia diagnosis was reported by Driessen et al. [29]. FFR-CT was excellent in diagnosing vessel-specific ischemia in a total of 208 patients who were investigated for CAD. Additionally, Anagnostopoulos et al. [30] tested the relationship of CCTA-based vFAI with regional flow parameters derived by quantitative PET and its utility in determining the abnormal vasodilating capability of coronary vessels with stenotic lesions at CCTA. In 78 patients, the vFAI, stress myocardial blood flow (MBF) and myocardial flow reserve (MFR) were estimated. The CCTA-based vFAI was positively correlated with the stress MBF (R = 0.49, *p* < 0.001 and R = 0.53, *p* = 0.001) and with the MFR (R = 0.41, *p* < 0.001 and R = 0.39, *p* = 0.004) for 15O-water- and 13N-ammonia-based measurements, respectively. The accuracy of the vFAI for predicting the abnormal stress MBF in 15O-water studies was like that of CCTA. However, the vFAI performed better than CCTA in predicting the abnormal MFR. For 15O-water PET studies, the per-vessel specificity and sensitivity was 90.9% and 77.8%, respectively, for predicting a stress MBF ≤ 2.3. For 13N-ammonia, the per-vessel sensitivity was 100%, and the specificity was 76.9%.The outcome point revealed that when the vFAI is combined with anatomical data, the diagnostic accuracy of CCTA is higher. In summary, compared with the offsite studies, the major advantage of the onsite calculation of the FFR is the reduction of logistic expenses and the vital time saved when it is applied on an urgent basis. In addition, smaller expenses might result in a more well-known software for functional lesion estimation. The disadvantages include the fact that there is no independent control over the CCTA image quality, which has been identified as a decisive factor influencing the results of the CT-derived FFR (Figure 3). 

### 3.3. FFR-CT and the Impact on the Decision-Making Process

Lately, there have been many studies that explored the clinical endpoints of virtual FFR-CT-guided diagnostic formations compared to usual practice in suspected high-risk patients and provided a more resource-centric approach to the discussion about FFR-CT and its clinical utility [13].The clinical efficiency of a model using FFR-CT to guide decision-making, compared with conventional testing, had been demonstrated in the PLATFORM (Prospect Longitudinal Trial of FFR-CT: Outcome and Resource Impact) trial [31]. The aim of this research was to measure the quality of life and financial results of evaluation strategies that use FFR-CT. A total of 584 patients with referring angina were assigned in prospect to perform either the usual testing (n = 287) or CCTA/FFRCT (n = 297). The primary endpoint of this study was based on the percentage of those with planned ICA in whom no significant obstructive CAD (no stenosis ≥50% by core laboratory quantitative analysis or invasive FFR ≤ 0.80) was found at ICA within 90 days. A second endpoint of this study included death, myocardial infarction and unplanned revascularization, which were independently and blindly adjudicated. CCTA/FFR-CT was related to a notably lower rate of ICA showing no obstructive CAD. The recently completed PROMISE [32] and SCOT-HEART [33] trials suggested that an evaluation strategy based on CCTA enhances the diagnostic performance, improves the efficiency of triage to invasive catheterization, and may reduce the radiation exposure in comparison with functional stress testing, and it may lead to similar rates of cardiac events [34]. They showed that reserving ICA for patients with an FFRCT of ≤0.80 could decrease ICA without ≥50% stenosis by 44%, and increase the proportion of ICA leading to revascularization by 24%. Since the rate of coronary revascularization in the PROMISE was doubled by CCTA as opposed to functional testing, this issue is a significant consideration to take into account. The recent FFRCT RIPCORD study [35] evaluated the impact of FFRCT and demonstrated that the availability of FFRCT results had a substantial effect on the labeling of significant CAD and management of patients compared to coronary CCTA alone. The HeartFlow technology was used for the extraction of FFRCT and 200 patients from the NXT trial were included. Alternation was noticed in the designated executive category compared to CCTA alone in 72 cases (36%) when the post-FFRCT data were reported. A useful software for decision-making support for management of cases with CAD based on reconstruction of atherosclerotic plaque process was developed in the SMARTool system [28]. This was achieved by performing computational modeling of the major process of the atherosclerotic plaque growth. Firstly, a CCTA was acquired and then 3D modeling of the arterial trees was performed. Then, plaque development modeling and boundary conditions were employed to simulate the primary procedure of atherosclerosis (e.g., estimation of ESS). The plaque growth model was based on both biological and genetic data of the patient. Also, through this system, the extraction of the vFAI was achieved. Finally, the modeling of the stent deployment was performed. The aforementioned were integrated into a cloud platform, which provides a decision support tool to doctors for stratification, diagnosis, prediction and treatment to promote personalized medicine. The development of FFR-CT and its utilization in this condition offers the efficacy to reduce unnecessary invasive testing and improve outcomes, owing to its notable precision and its unique ability to detect prognostically important but non-obstructive CAD [28]. Clinical interpretation of FFR-CT in conjunction with anatomic assessment of CAD by CCTA is dependent on appropriate coronary luminal modeling. Inadequate signal or contrast relative to noise and coronary motion or misalignment of artifacts may compromise the ability to conduct FFR-CT analysis. Efforts to improve the standardization of reporting are crucial in order to realize the chance of FFR-CT positively influencing the clinical supervision of individuals suffering from coronary artery disease (Table 1).

## 4. Virtual FFR Based on Invasive Imaging 

### 4.1. Invasive Angiography Derived Virtual FFR

#### 4.1.1. Early Pioneering Studies

FFR computation derived from angiographic imaging data alone reveals as the logical extension of the extra information that conventional angiography can provide when a patient arrives at the cath-lab and has the advantage of better image resolution compared to the CCTA. The first in vivo study to present accurate CFD from coronary-based FFR measurements in the coronary circulation was VIRTU-1 (VIRTUal Fractional Flow Reserve Angiography) [36], which included 19 patients with chronic coronary artery disease awaiting elective percutaneous intervention. The computer model they used for virtual FFR assessment from rotational angiographic images was quite complex and required an average time of 24 h for analysis. The comparison against the invasive FFR showed a significant correlation, while the precision for detecting an FFR ≤ 0.8 was 97%. FFR-QCA was presented as a computer model for the quick estimation of the FFR in lesions with intermediate coronary stenoses [37]. The approach was to create anatomic reconstruction models by 3D-QCA and then apply CFD using the hyperemic flow rate derived by 3D QCA and the TIMI frame count as boundary condition. Computation of FFR-QCA was studied in 68 patients (77 patients). Every vessel analysis lasted less than 10 min. This model, however, required the use of vasodilators for causing hyperemia (to calculate the hyperemic volumetric flow rate) during ICA. The mean stenosis diameter was 46.6 ± 7.3% and FFR-QCA correlated well with the FFR (r = 0.81, *p* < 0.001). A fast computational model for assessing the virtual functional assessment index (vFAI) in intermediate coronary lesions based on routine angiographic images without applying induced hyperemia was proposed by Papafaklis et al. [38]. This virtual index was proposed as a reliable alternative to FFR assessment in patients who underwent ICA. In a total of 139 vessels (120 patients) with intermediate lesions assessed by wire-FFR (reference standard: ≤0.80), 3D QCA was performed. The proposed approach used a 3D-QCA model and steady-flow CFD algorithm to calculate the vFAI, which was defined as the ratio of distal to proximal pressure over the lesion for flows in the range from 0–4 mL/s, normalized by the ratio over this range for a normal artery. The discriminative ability of the vFAI for ischemia-producing lesions was high (area under the receiver operator characteristic curve [AUC]: 92% [95% CI: 86–96%]). The vFAI was superior to standard 3-D QCA in discriminating an FFR ≤0.80 (AUC: 78% [95% CI: 70–84%]; *p* < 0.0001 compared to vFAI). High specificity and sensitivity (88, 90, and 86%, respectively) in predicting an FFR ≤0.80 were described and there was a narrow correlation (r = 0.78, *p* < 0.0001) and concurrence of the vFAI in contrast to wire-FFR (average difference: −0.0039 ± 0.085, *p* = 0.59). The mean total time required for this procedure (3D-QCA and CFD modeling) was nearly exactly 15 min per vessel using an off-the-shelf workstation. The prospective multicenter FAVOR Pilot Trial study presented a new virtual tool, namely the quantitative flow ratio (QFR) [39]. To minimize the computation time, instead of CFD techniques, simplistic fluid dynamic equations were applied based on early experimental reports of the flow through single arterial stenosis models. The FAVOR study (84 vessels in 73 patients with moderate coronary lesions) aimed to identify the finest way to use this tool, investigating the offline computation of the QFR compared with conventional pressure wire-based FFR. Three different blood flow models (volumetric flow input conditions) were tested for the calculation of the QFR. The first aims at a hyperemic flow velocity of 0.35 m/s (fQFR). On the other side, the cQFR predicts the hyperemic contrast-flow velocity by using the TIMI frame count from standard coronary angiographic images. Finally, according to the third one, the hyperemic flow velocity could be derived by the TIMI frame count while adenosine is infused. In routine clinical work, the cQFR was recommended as an index. Although physicians have to manually interact, the total cQFR in-operation computation time was reported as 5 min on average. Good correlation rates with the FFR were noticed for the fQFR, cQFR, and aQFR (r = 0.69 [*p* < 0.001]; r = 0.77 [*p* < 0.001]; r = 0.72 [*p* < 0.001], respectively). Also, the accuracy of diagnosis for determining an FFR ≤0.80 in a per-vessel analysis was the highest for the aQFR (87%; 95% CI: 80 to 94%), followed by the cQFR (86%; 95% CI: 78 to 93%) and fQFR (80%; 95% CI: 71 to 89%).

#### 4.1.2. Large Clinical Studies (QFR, FFRangio)

After the early FAVOR pilot study, the QFR was observed to have good diagnostic accuracy and correlation with the FFR in larger prospective in-procedure, retrospective and prospective offline studies. The Functional Assessment by Virtual Online Reconstruction (FAVOR) II trials went through a head-to-head comparison of the high diagnostic accuracy of the in-procedure QFR diagnostic performance using the FFR as a reference standard [39,40]. The FAVOR II China study (308 patients enrolled in 5 centers) was the trial to measure the diagnostic precision of the QFR [40,41]. Online analysis of the vessel-level QFR had a diagnostic accuracy of 92.7%, and offline analysis of the vessel-level QFR had a high diagnostic accuracy of 93.3%. The FAVOR III Europe and Japan study proved the superior specificity and sensitivity of the QFR for the detection of functionally severe lesions when compared with 3D-QCA using the FFR as the reference standard in a sample of 2000 patients. An advantage of the QFR was the time for the analysis compared to the invasive measurement of the FFR. The comparison between the QFR and FFR showed a notable variation in time [4.8 min (IQR 3.5–6.0) versus 7.0 min (IQR 5.0–10.0)]. The WIFI II study [42] (Wire-Free Functional Imaging II) was a predefined sub-study of the Dan-NICAD study (Danish Study of Non-Invasive Diagnostic Testing in CAD) with the main target point being the estimation of the feasibility and performance of the QFR. The FFR was performed in 292 lesions, and the QFR was calculated in 240 lesions. The median QFR was 0.84 (IQR, 0.77–0.89) and there was a major association (r = 0.70, *p* < 0.0001) and precision (mean difference of 0.01 ± 0.08, *p* = 0.08) compared with the FFR. The QFR diagnostic accuracy was in the range of 0.77 to 0.83 (83–87%; *p* = 0.002), i.e., around the diagnostic cut-off point. The average time to calculate the QFR was 266 s (IQR 181–332 s), including the time for two optimal angiographic acquisitions and QFR calculation [43]. While the clinical interest in the development of a software (Art care, version 1.0) [44] that provides both 3D reconstruction and functional assessment of the coronary arterial tree increases, a state of the art software was developed by Siogkas et al. [44]. This platform provided to clinicians the ability to reconstruct the desired 3D vessel using different imaging modalities (fusion of IVUS and ICA, fusion of OCT and ICA or just ICA). Furthermore, one of the main points of this study was the use of a dedicated method for the calculation of the vFAI for the reconstructed model. Thus, it offered both anatomic and functional assessment of the culprit vessel. For the validation of this study, a comparison process with a commercial reconstruction software (CASS QCA 3D^®^, version 1.0) was implemented using different metrics, such as the volume of the 3D model, the length and the minimum lumen diameter (MLD) as well as the calculated vFAI on eleven coronary arteries. A high connection was noticed among the two methods, with Pearson’s correlation coefficient (R) measured at 0.99, 0.99, 0.88 and 0.99, respectively. In addition, the automatic lumen identification modality for IVUS and OCT showed a high accuracy compared to the annotations by cardiology experts. In comparison to other publicly available software, the proposed system offers several advantages since it is the only one that can utilize all three coronary imaging modalities. Depending on the available modality, different full 3D reconstructions and the ensuing vFAI calculations require different amounts of time (2 min for the 3D-QCA module, 10 min for the IVUS-ICA and OCT-ICA modules). Another method, namely FFRangio, has been recently presented for estimating the FFR without the use of a coronary pressure wire or hyperemic agent. This model is derived from the 3D coronary artery tree reconstruction and the estimation of the resistance and flow across the stenosis. FFRangio is calculated as the ratio of the maximal flow rate in the culprit artery compared with the maximal flow rate in the lesion without stenosis, and it was equated with the FFR at the exact point of the pressure wire sensor [44]. A multicenter international trial, the FAST-FFR study [45], was conducted with the assignment of contrasting the accuracy of onsite FFRangio with pressure wire-FFR. For each patient, coronary angiography was applied and coronary pressure wire-FFR was measured by operators blinded to FFRangio. Overall, FFRangio’s diagnostic accuracy was 92%, and it was noteworthy that this number held true when focusing solely on FFR values between 0.75 and 0.8. Although the virtual assessment of the FFR throughout the entire coronary tree is a significant advantage that would assist the widespread physiological coronary lesion estimation, it is not clear how much is the total time required to calculate FFRangio, including the manual processing time for the entire coronary tree. Witberg et al. [46] analyzed 5 prospective cohorts with 700 lesions from 588 patients (mean age 65 years, 71% men, 40% with acute coronary syndromes) in which FFRangio was compared against the reference standard wire-based FFR. The FFRangio yielded favorable diagnostic performance, with an accuracy of 93%. The increased diagnostic accuracy was also consistent among the different subgroups under investigation. Moreover, the C-statistic for the FFRangio was 0.95, which further highlights its discriminatory power, which is important in clinical practice. More recently, Siogkas et al. [27] introduced a novel hemodynamic index for coronary stenosis, SmartFFR, which was compared with the gold standard FFR. In this study, the SmartFFR was calculated after the 3D reconstruction of the vessel and the blood flow simulation based on a dataset of 98 patients (114 arteries). A small overestimation of the FFR by this index on this occasion gave an average difference of 0.024 ± 0.051 (*p* < 0.0001), where a high correlation was noticed in the diagnostic performance of the proposed index using the established FFR threshold 0.80 (AUC = 0.975, *p* < 0.001).

#### 4.1.3. Outcome-Based Studies

**Figure 4:**
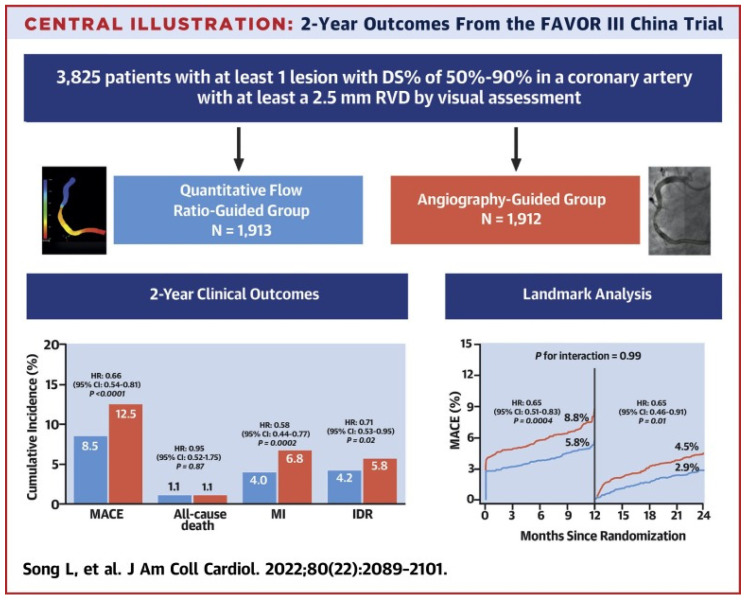
Study flowchart [50]. This figure is adapted from Song et al. 2022 [47] with permission of Copyright Clearance Center’s RightsLink.

Beyond studies assessing the correlation and diagnostic accuracy of the virtual indices compared to the FFR, the next big step would be outcome-based randomized trials. A multicenter blinded, randomized, sham-controlled trial (FAVOR III China) aimed to establish whether a PCI strategy based on the QFR compared to the usual strategy based on angiography for decision-making could yield better results. In FAVOR III China [47], the patients (3847) and all post-interventional specialists and researchers were screened for randomization allocation. In order to control participant masking, patients in both groups wore music-playing headphones during the process and had a preset 10 min delay for real or sham QFR calculation before PCI. A masking questionnaire was delivered to every discharged patient, and at 6 months and 2 years post-procedure, to estimate the achievement of randomization concealment and the perception of treatment allocation. Among patients undergoing PCI, a QFR-guided strategy of lesion selection improved the 2-year clinical outcomes (myocardial infarction, death from any cause, or ischemia driven revascularization) compared with standard angiography guidance. Patients whose QFR assessment altered the intended revascularization strategy benefited the most (HR: 0.66; 95% CI: 0.54–0.81; *p* < 0.0001), driven by fewer MIs (4.0% vs. 6.8%; HR: 0.58; 95% CI: 0.44–0.77; *p*¼ 0.0002) and ischemia-driven revascularizations (4.2% vs. 5.8%; HR: 0.71; 95% CI: 0.53–0.95; *p*¼ 0.02). In the randomized PANDA III trial [48], the QFR was retrospectively analyzed from the angiograms of 1391 patients who were grouped into those having had QFR-consistent treatment (all functionally ischemic vessels [baseline QFR ≤ 0.80] were treated and all non-ischemic vessels [baseline QFR > 0.80] were deferred) and those having had QFR-inconsistent treatment. QFR-consistent PCI was performed on 814 (58.5%) patients overall, whereas QFR-inconsistent PCI was performed on 577 (41.5%) patients. The risk of 2-year MACE was lower in patients receiving QFR-consistent treatment as compared to those receiving QFR-inconsistent treatment (8.4% vs. 14.7%; hazard ratio [HR] 0.56 [95% CI, 0.41–0.78). About 60% of patients in this post hoc analysis of an all-comers PCI trial received treatment in line with what a QFR measurement would have suggested, and achieving this was linked to better 2-year clinical outcomes. Lately, an innovative multicenter randomized trial, FAST III, explored in approximately 2228 patients with moderate coronary artery stenosis the relation of vFFR-guided versus FFR-guided revascularization in terms of the clinical outcomes at the 1-year follow-up (all-cause death, any myocardial infarction (MI), and any revascularization within 1 year). Intermediate lesions are physiologically assessed using on-line vFFR or FFR and treated if the vFFR or FFR is ≤0.80. The virtual FFR was calculated with the CAAS software (CAAS Workstation 8.5, Pie Medical Imaging, Maastricht, The Netherlands) by certified operators who followed a dedicated training program. The non-inferiority of vFFR-guided revascularization is declared if the upper boundary of the 95% CI of the rate difference of the primary end point falls below 3.0% [49] (Figure 4).

#### 4.1.4. Discrepancy Versus FFR

Observational data reveal more discordance between the QFR and FFR in patients with previous MI, diabetes, kidney disease, severe stenosis (high percentage diameter or long lesion length stenosis), and severe aortic stenosis (aortic valve area < 0.60 m^2^). However, the entire validation exported data appeared comparable and promising. In addition, heterogeneous results were compared to non-invasive imaging with the QFR. An elevated distal microvascular resistance and impaired ability to dilate the microvasculature could be involved in the reported QFR vs. FFR discordance rate observed in patients with diabetes, previous MI, and microcirculatory dysfunction. However, it is uncertain which index imports a “real” calculation of the epicardial lesion severity in the setting of increased microvascular resistance, because the FFR is inherently affected by microvascular dysfunction.

### 4.2. Virtual FFR Based on Intravascular Imaging

Efforts to examine the accuracy of virtual functional assessment of coronary lesions using 3D coronary artery reconstruction based on intravascular ultrasound (IVUS) against the invasively measured FFR have been recently reported. A software platform presented by Siogkas et al. offered clinicians the ability to calculate the vFAI [50] through CFD simulations based on 3D models derived by the fusion of IVUS and ICA in a short time. The derived 3D model was represented and then applied to blood flow simulations, resulting in the calculation of the vFAI. Seike et al. [51] retrospectively analyzed 50 lesions in 48 patients with coronary stenosis who underwent IVUS and FFR at the same location. The metric called the IVUS-FFR was calculated using an algorithm, which was based on a simplified equation (Poiseuille resistance). The average percent diameter stenosis detected on QCA and the mean FFR were 56.4 ± 10.7% and 0.69 ± 0.08. The IVUS-FFR had a higher linear association with the FFR (R = 0.78, *p* < 0.001) than the IVUS-derived minimum lumen area (MLA) had (R = 0.43, *p* = 0.002). The IVUS-FFR was estimated based on normal coronary circulation and many disorders such as hypertrophic cardiomyopathy, LV hypertrophy and valvular disease were absent. Therefore, to precisely evaluate patients with these disorders, further investigation is needed. Another pilot study by Siogkas et al. [50] investigated the feasibility and diagnostic performance of the IVUS-based vFAI against the FFR. Twenty-two patients underwent IVUS and FFR, with five patients presenting an FFR ≤ 0.80. The obtained IVUS-based geometries were processed with CFD techniques to calculate the vFAI, as previously presented. As a result, great concordance among the IVUS-based vFAI and FFR was noticed. The proposed method provides physiologic and anatomic data, as a result enabling complete and comprehensive estimation of coronary vessels pre- and post-intervention using an intravascular imaging catheter without requiring the pressure wire. OCT is used for anatomic and morphological assessment of coronary lesions and provides lumen measurements with excellent reproducibility. OCT also provides information about plaque vulnerability, calcification, and other parameters, which helps in guiding the procedure along with diagnostics [52]. These particular properties were used by Art care [44], a multimodality software from which the vFAI index was calculated using the 3D models derived from the fusion of OCT and ICA. The luminal and outer borders are automatically annotated in the OCT frames, then the software utilizes the luminal borders from the centerline extraction module and creates the respective contours for the final 3D model. The 3D model created can be subjected to computational blood flow simulations, with the material properties for blood (i.e., density and viscosity) having been defined by the user. Finally, the required blood flow simulations were performed in order to calculate the vFAI. Zafar et al. [52] investigated the potential of the OCT-derived FFR for the estimation of culprit coronary lesions (stenosis were labeled severe if FFR ≤ 0.8). The thesis of this study was to assess the blood flow rate and velocity in coronary tree stenosis, calculated through the volumetric analysis of frequency domain optical coherence tomography (FD-OCT) pull back images of the vessel segments, and investigate the correlation between the FD-OCT extracted measurements and FFR. This study contained a total of 26 coronary stenoses in 20 patients with stable angina and/or ischemia documented on an exercise stress test that were studied consecutively with QCA, pressure derived FFR, and OCT during diagnostic coronary angiography. There was an intermediate but significant matching among the pressure-derived FFR and OCT-derived FFR (r = 0.69, *p* < 0.001). The Bland–Altman report revealed that the average differences between the pressure-derived FFR and OCT-derived FFR were 0.05 ± 0.14 (limits of agreement: −0.09 to 0.19). The variation in the FFR between the pressure-derived FFR and FD-OCT-derived FFR was found to have a root mean square error (RMSE) of 447 ± 0.087 FFR units. The OCT-derived FFR has the probability to become a valid tool for the evaluation of coronary artery stenosis. In a larger study performed by Ha et al. [53], 92 patients with moderate lesion stenosis in the left anterior descending artery received both FFR assessment with OCT performance and pressure wires. The computational FFR calculation was achieved by a CFD algorithm based on OCT data (FFR-OCT). FFR-OCT had 88% diagnostic accuracy using the wire-based FFR 0.80 cut-off as a reference. FFR-OCT also had a stronger correlation with the FFR measurements (r = 0.89, *p* < 0.001) than QCA-percent diameter stenosis (r = −0.65, *p* < 0.001) and OCT measurements of minimum lumen area (r = 0.68, *p* < 0.001) and percent area stenosis (r = −0.70, *p* < 0.00). Wei Yu et al. [54] presented a novel method for estimating the FFR from OCT (OFR). A total of 143 vessels from 135 patients were analyzed in the catheterization laboratory. The analysis included patients who underwent both OCT and FFR prior to intervention. The OFR at each position along the culprit vessel was computed based on a validated method derived from a computational FFR model, applying a virtual volumetric flow rate at the inlet boundary. The hyperemic volumetric flow rate was computed by multiplying the proximal reference lumen area from the OCT by a virtual hyperemic flow of 0.35 m/s. Following analysis, the calculated OFR values were used to color-code the reconstructed artery, and the OFR value at the most distal point was used to compare it to the FFR. On an off-the-shelf workstation, the average reconstruction time from the point at which the OCT image pullback was entered into the software program until the OFR computation was completed was 55 ± 23 s. The per-vessel diagnostic accuracy of the OFR was 90% (95% CI: 84% to 95%) using the FFR as the gold standard method. The OFR has potential for optimizing complex PCI procedures. In addition, the OFR is not reliant on angiographic imaging, which could be challenging to identify in complex coronary anatomy. Furthermore, the shorter and more automated analysis time for the OFR is also suitable for routine use in the laboratory. The fusion of angiographic and OCT data has also been used as the basis for virtually deriving the FFR [54]. The pulsatile non-Newtonian coronary flow was simulated using CFD techniques to virtually derive the FFR [55]. The virtual FFR could also be co-registered along the OCT pullback segment, providing a screenshot of the vFFR that was positively associated with the beneficial characterization of the plaque burden (Table 2). 

## 5. Functional Angiography and Coronary Imaging: Future Perspectives

During the last decade, fluid mechanics algorithms and CFD techniques have had widespread application in the virtual evaluation of moderate coronary stenosis using both invasive and non-invasive coronary imaging. A large number of validation studies have been performed and have shown that virtual indices have a strong correlation with the pressure-wire derived FFR. Newer methods have overcome the obstacle of the offsite analysis and have allowed for a faster onsite calculation, presenting a new era of functional angiography. Could the virtual functional tools replace the pressure-wire derived FFR? Several studies have shown high but not optimal correlations versus the gold standard. Therefore, additional research is required in order to validate the invasive and non-invasive methods for the virtual functional assessment of coronary arteries using clinical outcome studies in patients with simple and complex CAD. The pitfalls of the present methodologies are not uncommon and need future improvements. Despite the limitations mentioned, FFR-CT could potentially become the most efficient gatekeeper for invasive investigations, providing not only a reliable 3D reconstruction of coronary anatomy but also a complete hemodynamic assessment of the coronary tree. The angiography-derived FFR provides a lumenogram with higher spatial resolution than CCTA, but ICA is not free from pitfalls. Further research is needed in order to validate the clinical potential of the angiography-derived FFR to provide a quick (i.e., online; at the time of catheterization) and reliable alternative to the FFR, obviating the need for the pressure-wire and drug-induced hyperemia. The latest randomized trials showing that a QFR-guided PCI strategy resulted in a superior clinical outcome compared to the standard angiography-guided PCI provide data for consideration within the context of the guidelines.

## 6. Conclusions

The FFR is the gold standard for the detection of ischemia-inducing coronary stenosis. Improved clinical outcomes and the reduction of repeat revascularization are associated with FFR-guided PCI. Virtual functional assessment acquired by coupling different imaging techniques with fluid mechanics algorithms or CFD models is an attractive alternative to the invasive FFR. Various studies during the last decade have presented hemodynamic indices derived from both invasive and non-invasive imaging, which showed great correlation against the pressure-wire derived FFR. New algorithm-based fluid models have been introduced and contribute to a path toward patient-tailored treatment strategies that are based on the combination of physiology and anatomy for complete and comprehensive assessment of coronary stenosis. However, numerous scientific and logistical pitfalls must be overcome in order to enter into our routine clinical practice these computational approaches.

## Figures and Tables

**Table 1:** Virtual FFR based on non-invasive imaging.

Study, Year	Imaging Modality	Sample Size (Patients, Vessels)	Pearson Correlation Coefficient	Agreement (Bias ± SD: Virtual Index−FFR)	Overall Diagnostic Accuracy	AUC
DISCOVER-FLOW, 2011 [14]	CCTA	103, 159	0.68	0.02 ± 0.116	84% (per vessel)	0.90
DeFACTO, 2012 [15]	CCTA	252, 407	0.63	0.06	73% (per vessel)	0.81
HeartFlow NXT, 2014 [16,17]	CCTA	251, 484	0.82	0.02 ± 0.074	86% (per vessel)	0.93
Renker., 2014 [20]	CCTA	53	0.66	-	77%	0.92
Kruk, 2016 [21]	CCTA	90, 96	0.67	−0.01 ± 0.095	74%	0.83
Ko, 2016 [22]	CCTA	42, 78	0.57	−0.065 ± 0.137	83.9% (per vessel)	0.88
Donnelly, 2018 [23]	CCTA	44	0.73	−0.48	63%	0.89
Röther, 2018 [24]	CCTA	71, 91	0.85	0.0049	86%	0.94
Wardziak, 2019 [25]	CCTA	90, 92	-	-	74%	0.835
Siogkas, 2019 [26]	CCTA/vFAI	63, 74	0.89	0.034 ± 0.042	93.2%	0.97
Anagnostopoulos, 2019 [30]	PET/CCTA	78	0.79	-	78.6% and 75% (MBF and MFR) (^15^O-water)	0.866 and 0.737 (MBF and MFR) (^15^O-water)
					82.7% and 71.2% (MBF and MFR) (^13^N-ammonia)	0.887 and 0.78 (MBF and MFR) (^13^N-ammonia)
Siogkas, 2021 [27]	SmartFFR CCTA/ICA	167, 202	0.83	0.007 ± 0.053	86.4%	0.956

AUC = area under the curve, CCTA = coronary computed tomography angiography, DeFACTO = Determination of Fractional Flow Reserve by Anatomic Computed Tomographic AngiOgraphy, DISCOVER-FLOW = Diagnosis of Ischemia-Causing Stenoses Obtained Via Noninvasive Fractional Flow Reserve, FFR = fractional flow reserve, HEARTFlow NXT = HeartFlow NXT-Heart Flow Analysis of Coronary Blood Flow Using Coronary CT Angiography: NeXt-sTeps, ICA = invasive coronary angiography, MBF = stress myocardial blood flow, MFR = myocardial flow reserve, PET = positron emission tomography vFAI = virtual functional assessment index.

**Table 2:** Virtual FFR based on invasive imaging.

Study, Year	Imaging Modality	Sample Size (Patients, Vessels)	Pearson Correlation Coefficient	Agreement (Bias ± SD: Virtual Index—FFR)	Overall Diagnostic Accuracy	AUC
Morris et al. (VIRTU-1), 2013 [36]	Invasive angiography	19, 35	0.84	0.02 ± 0.080	97% (per vessel)	0.97
Tu et al., 2014 [37]	Invasive angiography	68, 77	0.81	0.00 ± 0.06	88.3%	0.93
Papafaklis et al. (vFAI), 2014 [38]	Invasive angiography	120, 139	0.78	0.004 ± 0.085	87.8%	0.92
FAVOR Pilot Study (QFR), 2016 [39,40,41]	Invasive angiography	73, 84	fQFR: 0.69	0.003 ± 0.068	80%	0.88
			cQFR: 0.77	0.001 ± 0.059	86%	0.92
			aQFR: 0.72	−0.001 ± 0.065	87%	0.91
FAVOR III Pilot Study (QFR), 2023 [39,40,41]	Invasive angiography	2000	0.70	−0.01± 0.063	92.7% (per vessel)	0.96
WIFI II study (QFR), 2018 [42]	Invasive angiography	191, 292	0.70	0.00±0.06	83%	0.86
FAST-FFR (FFRangio), 2019 [44,45]	Invasive angiography	301, 319	0.80	−0.14 to 0.12	92%	-
FAST-FFR III (FFRangio), 2023 [44,45]	Invasive angiography	2228	0.89	0.0029 ±0.0642	95%	0.93
Witberg et al. (FFRangio), 2021 [46]	Invasive angiography	588, 700	0.83	0.00 ± 0.12	93%	0.95
Seike et al. (IVUS-FFR) 2018 [51]	Intravascular Ultrasound	48, 50	0.78	0.057		
Siogkas et al., 2018 [50]	Intravascular Ultrasound	22	0.88	0.0196 ± 0.037	95.5%	-
Zafar et al., 2014 [52]	Optical Coherence Tomography	20, 26	0.69	0.05 ± 0.14	-	-
Yu et al., 2019 [54]	Optical Coherence Tomography	135, 143	0.70	0.01±0.07	87% (per vessel)	0.93

AUC = area under the curve, FAVOR III = Functional Assessment by Various Flow Reconstructions III, FFR = fractional flow reserve, IVUS = intravascular ultrasound imaging, QFR = quantitative flow ratio, vFAI = virtual functional assessment index, VIRTU-1 = VIRTUal Fractional Flow Reserve Angiography, WIFI = wire-free functional imaging.
